# Effect of the COVID-19 pandemic on emergency department attendances for pediatric injuries in Mozambique’s central hospitals: an interrupted time series and a comparison within the restriction periods between 2019 and 2020

**DOI:** 10.1136/tsaco-2022-001062

**Published:** 2023-07-14

**Authors:** Vanda Amado, Jette Moller, Maria Tereza Couto, Lee Wallis, Lucie Laflamme

**Affiliations:** 1 Department of Global Public Health, Karolinska Institute, Stockholm, Sweden; 2 Department of Surgery, University of Eduardo Mondlane Faculty of Medicine, Maputo, Mozambique; 3 Department of Community Health, University of Eduardo Mondlane Faculty of Medicine, Maputo, Mozambique; 4 Division of Emergency Medicine, University of Cape Town Faculty of Health Sciences, Cape Town, South Africa; 5 Institute for Social and Health Sciences, University of South Africa, Pretoria, South Africa

**Keywords:** COVID-19, pediatrics, Accidental Injuries, Emergency Treatment

## Abstract

**Objectives:**

Hospital-based studies indicate that restriction measures imposed during the COVID-19 pandemic have affected the number and characteristics of pediatric injuries. However, few studies have been conducted in resource-poor countries. This study aimed to determine whether injury-related emergency department (ED) attendances in Mozambique were affected during the restriction periods in 2020 and how the pattern of injury changed.

**Methods:**

Mozambique faced two restriction periods in 2020. An interrupted time series was applied to weekly data of pediatric injuries from the ED records of four central hospitals in Mozambique in 2019 and 2020. Weekly numbers of injuries were modeled using a Poisson regression model to estimate the effect of COVID-19 restrictions on trends over calendar time. Then, for each restriction period, differences in injury mechanisms, severity, need for surgery, and intensive care unit (ICU) attendances were compared between 2019 and 2020.

**Results:**

During the 76 weeks preceding the restrictions, there was a stable trend in ED attendances. The weekly number dropped by 48.7% after implementation of the first restrictions. By the end of 2020, the weekly numbers were back to the levels observed before the restrictions. Road traffic injuries (RTIs) and falls dropped during the first restriction period and RTIs and burns during the second. There was an increase of 80% in ICU attendances in all periods of 2020 at three hospitals during the first and second restriction periods.

**Conclusion:**

The COVID-19 restrictions yielded a reduction in the weekly number of pediatric injuries seen at Mozambique’s central hospitals, above all RTIs and falls. The drop reflects reductions in visits most importantly for RTIs, falls, and burns, but was accompanied by an increase in the proportion of ICU cases. This effect was not maintained when the restrictions were relaxed. Whether this reflects reduced exposure to injury or hesitancy to seek care remains to be determined.

**Level of evidence:**

Level III, retrospective study with up to two negative criteria.

**WHAT IS ALREADY KNOWN ON THIS TOPIC**

- The COVID-19 pandemic created many changes in how children lived their lives and how they were cared for by the healthcare systems, including hospital and emergency care.
- There is a vast body of knowledge on the pandemic’s impact on the burden, circumstances, and consequences of injuries.
- Most studies focused on the first months of the restrictions, were from different settings, and with mixed results.
- There have been few studies about childhood injuries and studies from low-income countries.

**WHAT THIS STUDY ADDS**

- In the specialized hospitals of Mozambique, the onset of the COVID-19 pandemic restriction measures was associated with an immediate, sharp, and significant reduction in emergency department (ED) attendances for pediatric injuries.
- After a sharp decrease, the overall number of pediatric injuries presenting to EDs increased progressively to reach the same level as before the onset of restrictions.
- By and large, although with variations between periods, a drop was found in the most common pediatric injury mechanisms during the restriction periods in 2020 (falls, road traffic injuries, and burns), which was typical of the largest hospitals, where the absolute differences were far bigger than in smaller hospitals where some absolute and relative increases were seen.
- Most strikingly, there was a remarkable increase in intensive care unit attendances.

**HOW THIS STUDY MIGHT AFFECT RESEARCH, PRACTICE OR POLICY**

- There might be within-country differences in how the pandemic affected the volume and pattern of emergency attendances for pediatric trauma care.
- In specialized hospitals, the periods during a pandemic may be characterized by absolute and relative reductions in the number of ED visits for pediatric injuries, with some differences depending on the size and site of the hospital.
- By contrast, cases seen may be more severe, which would put specific demands on the resources that are in place which are already burdened by pandemic-related issues.
- Whether the changes observed are attributable to a “true” reduction in the number of injuries resulting from less exposure due to the nature of the restrictions themselves, changes in referral practice, or hesitancy from the population to seek specialized care remains to be determined.

## Introduction

Injuries are a leading cause of mortality in children worldwide,^1 2^ and those who live in poverty, both within and between countries, are far more affected.^2 3^ Despite the extensive evidence available on how to prevent those injuries from happening or how to reduce morbidity and mortality, public health systems in low-income and middle-income countries (LMICs) struggle to reduce this burden^2 4^ and their healthcare services face several structural and organizational barriers.^5 6^ Such difficulties can be exacerbated during periods of a pandemic, where efforts are placed on containing the spread of the virus and providing care to infected individuals.^7^ A recent example is the COVID-19 pandemic,^8^ where several of the restriction measures implemented had a significant impact not only on the provision of healthcare services but also on people’s daily life and lifestyles,^9 10^ not least among children.^9 11–13^ For many of them, school closures, mobility restrictions, and reduced social interactions meant that their homes have become an environment where they would continue their studies and where they would interact to a far greater extent with siblings as well as with guardians who were also at home, either to work or because they were out of employment.^8 14^

Studies on pediatric populations from both high-income countries (HICs) and LMICs have reported that, in many instances, pandemic-related restrictions were associated with a reduction in certain types of injuries, such as road traffic injuries (RTIs)^15–17^ or sports and leisure injuries,^15 18–22^ and an increase in injuries that are more likely to occur in and around the home, such as burns^15 17 21–24^ and certain types of falls from heights.^19–21 25^ There are also indications of excess risk of violence-related injuries occurring at home, for example, injuries inflicted on children by either physical abuse and maltreatment^20 26–28^ or sexual violence.^29–31^ It is of note, however, that these differences are not consistent across studies and settings.^15 18 22 26 27 32 33^ Furthermore, in addition to changes in the number and epidemiological characteristics, studies have shown an increase in injury severity during the pandemic, whether viewed by the nature of the injuries,^15 21 25 33^ their severity,^16 21 22 25^ the provision of intensive care unit (ICU) treatment,^20 21 25 27^ or injury-related in-hospital mortality.^20^

To date, as is the case for various child health outcomes,^17^ studies on pediatric injuries from Sub-Saharan Africa (SSA) during the pandemic have been scarce.^7 34–36^ Increased knowledge from this region is warranted given the fact that, driven by the increasing level of RTIs and injuries related to interpersonal violence in the past decades, the western, southern, and central regions of SSA are the only three regions of the world reporting a non-significant decline in age-standardized injury disability-adjusted life years.^37^ The few studies on the effect of the pandemic in the SSA region revealed the following: a decline in the proportion of pediatric trauma during the pandemic, as part of the overall pediatric surgical volume, in Burkina Faso, Ecuador, Nigeria, Zimbabwe, and Zambia^17 36^; and an overall reduction in pediatric emergency attendances and a marked significant reduction of 36.1% in injuries among those attendances in Cape Town, South Africa^7 35^ and Nairobi, Kenya.^34^ As the SSA region is broad, countries were differentially affected during the pandemic, and as the pediatric population dominates studies from more countries are needed to get a broader picture of what the pandemic’s impact entailed in the region.

This national study from Mozambique investigates the impact of the two consecutive waves of restriction periods in 2020 on pediatric injuries presenting to the emergency department (ED) of the country’s four specialized hospitals. We first assessed the impact of the restrictions from their onset until the end of 2020, from all hospitals and with all injuries aggregated. We then analyzed how the pattern of injuries changed in absolute and relative terms at each hospital during each restriction period, with a focus on injury mechanisms and indicators of injury severity.

## Methods

### Study design

To assess the magnitude of the impact of the pandemic on ED attendances, an interrupted time series (ITS) was employed using average weekly numbers of injuries admitted to the ED covering 2 years, from 2019 to 2020 (105 weeks). This allowed for a long and stable preintervention time series to adequately predict the effect of the restriction periods had the restrictions not occurred. The two restriction periods from 2020 were implemented across the country, from April 1 to September 6 and from September 7 to December 31, and were called, respectively, the “emergency state” (20 weeks) and the “public calamity state” (12 weeks). The study was based on ED data aggregated from the country’s four central hospitals, the only ones providing specialized pediatric injury care.

Second, to depict changes in injury patterns, the characteristics of the injury were compared, by hospital and with all hospitals aggregated, for three time periods in 2020: prerestriction, emergency state, and public calamity state, each compared with the same calendar time period in 2019. Focus was placed on injury mechanism and severity indicator (severe injury at triage; ICU care and surgery). Differences in each period between 2020 and 2019 were measured in numbers and proportions of change.

The restrictions implemented were as follows.^38^ During the emergency state, the government imposed severe confinement measures that included advice to stay at home, closing of schools, halting of cultural and leisure activities (eg, parks, sports, pools), limiting public transportation, and prohibiting public and private events. Infringement was considered a crime of disobedience, with penalties such as prison, fine, or community service.^38 39^ During the public calamity state, some restrictions were relaxed under certain conditions, for example, schools reopened, sports resumed, beaches were reopened to the public, and some public events were allowed.^38 39^

### Setting

Mozambique is a low-income country located in southeastern Africa with an estimated 31 million inhabitants in 2021 (2017 census projections), of which approximately 45% were children aged 0 to 14 years, with a median age of 17.6 years.^40^ The country has 11 provinces divided into three regions. The southern region includes Maputo City, Maputo Province, Gaza, and Inhambane; the central region includes the provinces of Sofala, Manica, Tete, and Zambezia; and the northern region includes the provinces of Nampula, Cabo Delgado, and Niassa. All four central hospitals offer pediatric injury care and are geographically spread over the country: Maputo Central Hospital in the south (the oldest and the largest); Beira Central Hospital and Quelimane Central Hospital in the center, in Sofala Province and Zambezia Province, respectively; and Nampula Central Hospital in the north.

Table 1 presents the number of beds and clinical staff that each of the central hospitals have had as of December 2020. Maputo Central Hospital, located in the capital, is the referral hospital in Mozambique. It has more beds in total, but proportionately has less pediatric beds than the central hospitals of Nampula and Beira. The number of clinical staff varies a lot between hospitals, but the proportion of staff at the ED is quite comparable, except for Quelimane Central Hospital (7.8%), which is a smaller hospital.

**Table 1:** Number of beds and clinical staff at the four central hospitals of Mozambique in 2020

Central hospital	Opening year	Beds			Clinical staff		
		Total	Pediatric		Whole hospital	Emergency department	
		n	n	% total	n	n	% total
Maputo	1910	1512	307	20.3	4000	225	5.6
Beira	1951	1020	129	25.7	1650	89	5.4
Quelimane	2016	643	104	16.1	746	58	7.8
Nampula	1968	607	192	31.9	1570	92	5.9

Mozambique reported the first case of COVID-19 on March 2, 2020,^39^ and by December 5, 2020 there were a total of 17 042 confirmed cases, with 144 deaths. Table 2 shows the distribution of cases by region where the hospitals belong. Maputo City had 8942 (52.5%) cases and 112 (78%) deaths, followed by Zambezia with 1082 (6.3%) cases and 3 (2%) deaths^41^ (see table 2). Table 2 also shows that there were differences in how accessible and ready the hospitals were to deliver oxygen, with nearly 90% of the Maputo City population living at about 30 min drive to the hospital and only 12% of the population in Zambezia, and with no more than 18% of the population living at a distance of 1 hour from the hospital.^42^

**Table 2:** Overview of the COVID-19 situation in the regions where the central hospitals are located, as of December 15, 2020

Region/province and population*		Hospitals ready to deliver oxygen (total + the central hospital)†	COVID-19 cases (2020, by province)‡		Driving distance (in minutes) to health facility with oxygen to treat patients with COVID-19 (% of population)†	
Region	Province		Cases	Deaths, n (%)	60 min	30 min
South 6 433 912	Maputo City 1 127 565	5 + Maputo Central Hospital	8946	112 (1.3)	89.1	88.9
Center 13 401 550	Sofala 2 528 442	Only Beira Central Hospital	527	1 (0.2)	26.4	21.5
	Zambezia 5 709 418	6 + Quelimane Central Hospital	1082	3 (0.3)	18.3	11.9
North 10 996 782	Nampula 6 335 121	5 + Nampula Central Hospital	665	6 (0.9)	27.6	23.2
Total 30 832 244	20		Total 17 042	144 (0.8)		

*http://www.ine.gov.mz/noticias/antigas/populacao-mocambicana-para-2021.

†Denhard *et al*^42^ and Junior *et al.*^50^

‡United Nations Mozambique.^41^

It is also of note that the year 2020 was one of the most challenging years for Mozambique, especially among children . The pandemic hit only 8 months after the tropical cyclones Idai and Kenneth and as the war was about to intensify in the north of the country,^39^ displacing 13% of the population. The migration of the population in May 2020 increased the COVID-19 community transmission in Cabo Delgado and Nampula. Restriction measures were hard to follow and access to healthcare was difficult due to shortage in health professionals and weak infrastructures.^39^

### Data collection

Data on injury were extracted from the records of each hospital’s ED by local clinicians who were trained by the first author; follow-up information was collected at the pediatric surgery, neurosurgery, pediatric orthopedic, and plastic surgery wards. The case report form used was aligned with the WHO’s Injury Surveillance Guideline,^3^ and data were collected using tablets and uploaded to the Open Data Kit.^43^

We includede all pediatric injuries registered in the ED records, aged 0 to 14 years and admitted to the ED for at least 12 hours, admitted to a hospital ward (pediatric surgery, neurosurgery, pediatric orthopedics, or plastic surgery), or admitted to the ICU. We excluded any pediatric patient readmitted for the same injury within 30 days.

### Data analysis

We hypothesized a decrease in the overall average weekly injury rates following the stringent restriction period, with a possible increase over time. We analyzed data from all hospitals aggregated (n=2153 cases), compiling the average weekly injury rates in all four central hospitals aggregated. We conducted ITS using a Poisson regression model, as described by Bernal *et al*.^44^ The expected average weekly injury rate during the restriction periods was projected based on the 65 weeks preceding the first restriction period (ie, 52 weeks in 2019 and 13 weeks in 2020 before the second restriction period). To assess the immediate effect of the restrictions, a dummy variable was modeled to indicate the start of the restriction period. To detect possible change in rate over time, an interaction term between the dummy variable and calendar time was created and alignmented during the week of the restriction period. Hence, the model specification allowed for detection of both an immediate effect following the implementation of the restrictions during the emergency state and a change in the linear trend over time when the two restriction periods follow one another. Coexisting factors for injury incidence include a seasonality pattern.^45^ To adjust for this in the Poisson regression model, we included Fourier transformations of calendar time.^45^

The mechanism of injury was coded into four values: fall, RTI, burn, and other (including bicycle accident, interpersonal violence, stab/laceration, animal bite, foreign body, building collapse, and explosion).

To assess changing pattern in injury severity, we used three measures: “injury severity” at triage (mild, moderate, or severe), determined by the data recorder at the time of presentation, where “mild” was considered as minor or superficial cuts or bruises; “moderate” was defined as requiring some skilled treatment (eg, fracture reduction, sutures, incision and drainage); and “severe” was defined as requiring surgical or ICU-level management (eg, polytrauma, shock, major burn injuries, severe traumatic brain injury) or cases involving a threatened limb. Surgery was differentiated between surgical treatment (interventions in an operating theater under general anesthesia) and conservative treatment (all other interventions).

All analyses were conducted using Stata V.16. For the ITS, we also used Jamovi models (V.2.3; 2022).

## Results

Table 3 presents the changes in ED attendances for pediatric injuries from 2019 to 2020, by hospital and with all hospitals aggregated, for the whole year and split by three pandemic-related periods in 2020. For all hospitals aggregated, there was a 16.6% reduction in the total number of pediatric injuries from 2019 to 2020. Overall reductions were relatively sharp at Maputo Central Hospital (−19.9%) and Beira Central Hospital (−23.8%), whereas both Quelimane Central Hospital and Nampula Central Hospital faced an increase in pediatric injuries (+18.4% and +7.3%, respectively). The reduction was more pronounced during the emergency state (−24.3%) than the public calamity state (−16.4%). It is of note that Nampula, the most populated region of the country, is in a region affected by an ongoing war that peaked in early 2019, which can contribute and explain the sharp increase observed from 2019 to 2020 during the “prerestriction” period.

**Table 3:** Percentage of change in the number of pediatric injuries treated in the four specialized hospitals, by year and restriction period (n=2153)

Central hospital	Year	No restrictions		Emergency state		Public calamity state		Total	
		n	%	n	%	n	%	n	%
Maputo	2019	113		251		243		607	
	2020	132	16.8	206	−17.9	148	−39.1	486	−19.9
Beira	2019	73		191		131		395	
	2020	62	−15.1	102	−46.6	137	+4.5	301	−23.8
Quelimane	2019	7		44		25		76	
	2020	15	+1.1	46	+4.5	29	+16.0	90	+18.4
Nampula	2019	38		24		34		96	
	2020	22	−42.1	33	+37.5	48	+41.7	103	+7.3
Total	2019	231		510		433		1174	
	2020	231	0	386	−24.3	362	−16.4	979	−16.6

**Figure 1:**
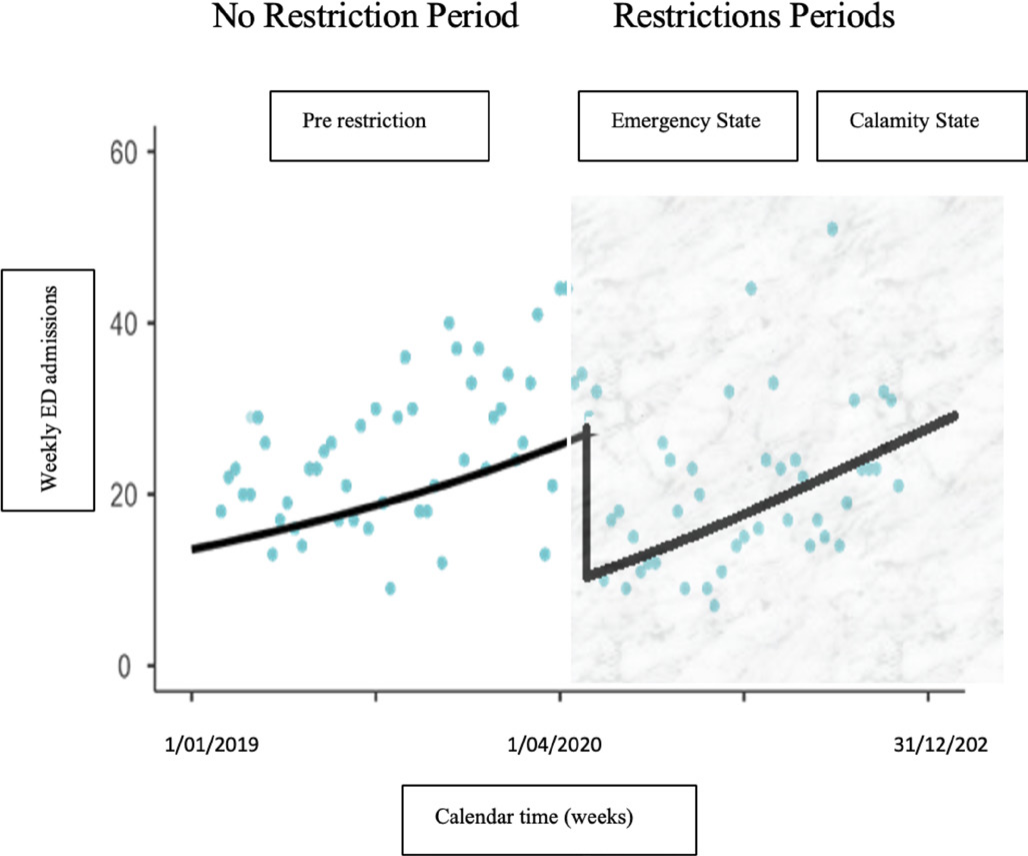
Weekly number of injuries from January 1, 2019 to December 31, 2020. There was a change in level (decrease in cases) immediately at the start of the restrictions (March 1, 2020; emergency state declared) and a gradual increase in slope. By the end of 2020 (public calamity state), the weekly number of injuries was similar to those observed before the restriction period. ED, emergency department.

Figure 1 presents the results of the ITS, for all hospitals and all injuries aggregated. It shows that, during the weeks from 2019 and 2020 preceding the onset of the restrictions, there was a relatively stable trend in pediatric injury ED attendances at an average of 17.6 injuries per week. After the onset of the two restriction periods, there was a sharp and sudden estimated decrease of 47.5% (CI 0.36 to 0.76) in the average weekly number of injuries. This sharp decrease did not last and was followed by a gradual increase over calendar time during the rest of 2020, with an overall number of ED attendances of about 16 injuries per week, a level close to the prerestriction period.

Table 4 presents the absolute and relative changes in ED attendances for pediatric injuries by injury mechanism, hospital, and restriction period, comparing the prepandemic year (2019) with the pandemic year (2020). With all injuries aggregated, from 2019 to 2020, there was a drop in the total number of admissions during each of the two restriction periods. This drop, however, was not observed for all injury mechanisms. During the emergency state, it was more evident for RTI and falls, with 69 and 45 cases less than in 2019 (−42.5% and −35.9%, respectively) and during the public calamity state (−33.6% for RTIs and −18.3% for burns). In the latter period, falls increased by about 3%.

**Table 4:** Absolute and relative changes in the number and proportion of cases by injury mechanism considering the hospital and the restriction period and comparing prepandemic with pandemic year (n=2153)

Injury mechanism	Prerestriction						Emergency state						Public calamity state					
	2019		2020		∆ 2020–2019		2019		2020		∆ 2020–2019		2019		2020		∆ 2020–2019	
	n	%	n	%	n	%	n	%	n	%	n	%	n	%	n	%	n	%
All central hospitals																		
Fall	127	55	104	45.1	−23	−18.1	192	37.6	123	31.9	−69	−35.9	171	39.5	176	48.6	+5	+2.9
RTI	60	26	50	21.7	−10	−16.7	106	20.8	61	15.8	−45	−42.5	107	24.7	71	19.6	−36	−33.6
Burns	21	9.1	51	22.1	+30	+142.9	178	34.9	179	46.4	+1	+0.6	109	25.2	89	24.6	−20	−18.3
Other	23	10	26	11.2	+3	+13	34	6.7	23	5.9	−11	−32.4	46	10.6	26	7.2	−20	−0.2
Total	231	100.0	231	100.0	–	–	510	100.0	386	100.0	−124	−24.3	433	100.0	362	100.0	−71	−16.4
Maputo Central Hospital																		
Fall	58	51.3	60	45.8	+2	+3.4	65	25.9	52	25.2	−13	−20.0	83	34.3	52	35.1	−31	−37.3
RTI	33	29.2	29	22.1	−4	−12.1	47	18.7	22	10.7	−25	−53.2	62	25.6	22	14.9	−40	−64.5
Burns	16	14.2	25	25.2	+18	+112.5	126	50.2	127	61.7	+1	+1	85	35.1	66	44.6	−19	−22.4
Other	6	5.3	9	6.9	+3	+50.0	13	5.2	5	2.4	−8	−61.5	12	5.0	8	5.4	−4	−33.3
Total	113	100.0	98	100.0	−15	−13.3	251	100.0	206	100.0	−45	−17.9	242	100.0	148	100.0	−94	−38.8
Beira Central Hospital																		
Fall	49	67.1	35	56.5	−14	−28.6	97	50.8	41	40.2	−56	−57.7	60	45.8	87	63.5	+27	+45
RTI	11	15.1	11	17.7	–	–	47	24.6	19	18.6	−28	−59.6	35	26.7	32	23.36	−3	−8.6
Burns	3	4.1	9	14.5	+6	+200.0	33	17.3	33	32.4	–	–	20	15.3	9	6.6	−11	−55.0
Other	10	13.7	7	11.3	−3	−30.0	14	7.3	9	8.8	−5	−35.7	16	12.2	9	6.6	−7	−43.8
Total	73	100.0	62	100.0	−11	−15.1	191	100.0	102	100.0	−89	−46.6	131	100.0	137	100.0	+6	+4.6
Quelimane Central Hospital																		
Fall	4	57.1	5	33.3	+1	+25.0	16	36.4	13	28.3	−3	−18.8	12	48.0	6	20.7	−6	−50.0
RTI	2	28.6	2	13.3	–	–	7	15.9	11	23.9	+4	+57.2	5	20.0	8	27.6	+3	+60.0
Burns	1	14.3	6	40	+5	+500.0	18	40.9	17	37	−1	−5.6	3	12.0	10	34.5	+7	+233.3
Other	0	0	2	13.3	+2	–	3	6.8	5	10.9	+2	+66.7	5	20.0	5	17.2	–	–
Total	7	100.0	15	100.0	+8	+114.2	44	100.0	46	100.0	+2	+4.6	25	100.0	29	100.0	+4	+16.0
Nampula Central Hospital																		
Fall	16	42.1	4	18.2	−12	−75.0	14	58.3	17	51.5	+3	+21.4	16	47.1	31	64.6	+15	+93.8
RTI	14	36.8	8	36.4	−6	−42.9	5	20.8	9	27.3	+4	+80.0	5	14.7	9	18.8	+4	+80.0
Burns	1	2.6	2	9.1	+1	+100	1	4.2	3	9.1	+2	+200.0	–	–	4	8.3	+4	–
Other	7	18.4	8	36.4	+1	+14.3	4	16.7	4	12.1	–	–	13	38.2	4	8.3	−9	−69.2
Total	38	100.0	22	100.0	−16	−42.1	24	100.0	33	100	+9	+37.5	34	100.0	48	100.0	+14	+41.2

∆, Delta symbol meaning "change" in the metric used. ; RTI, road traffic injury.

Although there were some similar changing patterns across hospitals, each of the hospitals had a specific pattern. In Maputo Central Hospital, during the prerestriction period, there were no remarkable absolute differences between 2019 and 2020, except for an increase in burns (n=18). During each of the restriction periods, there were absolute reductions in all injury mechanisms but one (burns during the emergency state), and the reductions were substantial, even more so during the public calamity state. The number of injuries in Beira Central Hospital dropped by 11 (15%) in 2020 during the prerestriction period, although there were more admissions for burns. Absolute numbers decreased even more during the emergency state (by 89 cases; 56 falls and 28 RTIs, among others), whereas they slightly increased during the public calamity state (n=6), an increase attributable to a large increase in falls (n=27), contrasting the reductions in all other mechanisms. Quelimane Central Hospital, the smallest one of all four hospitals, did not experience substantial drops during any period. On the contrary, the total number of cases increased in each period, yet the total and minor increases during the emergency state (n=2) are a result of reductions in falls and burns, contrasting the increase in RTIs and injuries of other mechanisms. Similar contrasts were also seen during the public calamity state, but the number of falls dropped and that of burns rose. In Nampula Central Hospital, the prerestriction period was marked by a reduction in the total case load by 16 cases (42.1%), whereas an increase occurred in the following two periods: a slight absolute increase in RTIs, falls, and burns during the emergency state, and again during the public calamity state, where falls increased by 15 cases (nearly doubled) and injuries of other mechanisms decreased by 9.

Table 5 shows how the numbers and proportions of the three measures of injury severity change between years and within periods. Overall, the numbers and proportions of severe injuries increased remarkably from 2019 to 2020 in the prerestriction period (mainly in Maputo Central Hospital, but also in Beira Central Hospital), but not during the two restriction periods, where by contrast there were overall moderate reductions. However, these hide period-specific differences between hospitals: for example, a reduction by 25 cases in Beira and an increase by 12 and 7 in Maputo and Nampula, respectively, during the emergency state, and a reduction in Maputo and an increase in Beira and Nampula during the public calamity state. For their part, overall admissions to ICU increased during both the prerestriction and the second restriction periods (above all due to an increase in Maputo Central Hospital and Beira Central Hospital, respectively). Further, during the prerestriction period, overall surgical treatment went slightly up, but with differences between hospitals: the number went up in Maputo only and down in Beira and Nampula. During the first restriction period, surgery went down again in Beira (n=−24), but only a handful of cases in Maputo (n=−2). During the public calamity state, there were no changes in the number and proportion of surgery treatment overall between 2019 and 2020.

**Table 5:** Change in the number of severe injuries admitted to the ICU or that received surgical treatment considering the hospital and the restriction period and comparing prepandemic with pandemic year (n=2153)

Specialized care	Prerestriction				Emergency state				Public calamity state			
	2019	2020	∆ 2020–2019		2019	2020	∆ 2020–2019		2019	2020	∆ 2020–2019	
	n	n	n	%	n	n	n	%	n	n	n	%
All central hospitals												
Severity	44	64	+20	+45.0	101	95	−6	−5.9	114	107	−7	−6.0
Admitted to ICU	8	15	+7	+87.5	31	56	+25	+80.6	11	24	+13	+118.2
Surgical treatment	60	51	−9	−15.0	78	52	−26	−33.3	78	78	–	–
Maputo Central Hospital												
Severity	17	30	+13	+76.5	21	33	+12	+38.5	61	35	−26	−42.6
Admitted to ICU	1	10	+9	+900.0	11	7	−4	−36.4	9	8	−1	−11.1
Surgical treatment	3	22	+19	+633.3	26	24	−2	−7.7	33	19	−14	−42.2
Beira Central Hospital												
Severity	16	25	+9	56.3	68	43	−25	−36.7	44	55	+11	+25.0
Admitted to ICU	4	4	–	–	16	1	−15	−93.8	2	11	+9	+450.0
Surgical treatment	21	13	−8	−38.1	42	18	−24	−57.1	34	36	+2	+5.9
Quelimane Central Hospital												
Severity	2	4	+2	+100.0	10	10	–	–	8	8	–	–
Admitted to ICU	–	–	–	–	2	2	–	–	–	3	+3	–
Surgical treatment	16	16	–	–	5	5	–	–	6	9	+3	+50.0
Nampula Central Hospital												
Severity	9	5	−4	−44.4	2	9	+7	+350.0	1	9	+8	+800.0
Admitted to ICU	3	1	−1	−66.7	2	4	+2	+100.0	–	2	+2	–
Surgical treatment	7	–	−7	−100.0	5	5	–	–	5	14	+9	+80.0

ICU, intensive care unit.

## Discussion

### Main findings

Our study shows that once the COVID-19 restrictions were implemented in the second quarter of 2020, there was a sharp reduction in ED attendances for pediatric injuries; however, this decrease was not maintained throughout the restriction periods. Rather, successively, there was a progressive increase in the weekly volume of injuries, and toward the end of 2020 the level reached was only slightly under the level before the restriction periods. When comparing each restriction period of 2020 with the same time period in 2019, our study also shows changes in the pattern and level of severity of the pediatric injuries, with some specificities by period and hospital.

The immediate drop seen in our ITS echoes what was observed in all-age and pediatric studies from HICs where, most often, proportions of injuries were compared before and during the pandemic. This applies to several studies from the USA,^15 16 20 27^ reporting reductions in overall volume of pediatric injuries of 13% and 19%,^15 16^ and for pediatric orthopedic injuries a 41% reduction.^20^ This was also seen in an Australian study showing an overall decrease in pediatric injuries by 15% during the country’s first lockdown, but not during the second one.^21^ Even greater reductions were observed in Spain in a multicenter study considering the first wave of restrictions, where compared with the same period of the previous year ED visits at three tertiary hospitals showed reductions in pediatric injuries of 83.5%, 75%, and 65.9% in the hospitals of Madrid, Valencia, and Palma de Mallorca, respectively.^46^

Our results are also in line with those of two previous studies in LMICs from SSA mentioned above. Indeed, one of them, a cross-country study, showed a reduction in the proportion of pediatric trauma as part of the overall pediatric surgical volume in four countries (Burkina Faso, Ecuador, Nigeria, and Zambia) during the pandemic,^17^ whereas the other one, limited to the Cape Town area in South Africa, showed a reduction in ED pediatric injury admissions in one healthcare facility.^35^

Many factors can contribute to the similarities and differences between studies. The first factor relates to the nature of the restrictions implemented,^7 9–11 14 17^ which differentially affected children’s exposure to hazards—the more confined the children were at an early stage, the less likely they were to be exposed to a range of hazards. This explains why in our study RTIs and falls decreased and burns increased more during the “state of emergency,” when more people were confined to homes and households were crowded. Falls increased in the second restriction period, during which restrictions were more relaxed. As parts of the northern region of the country were facing other issues, RTIs increased in Nampula Central Hospital and Quelimane Central Hospital during the emergency state, eventually less than they could have done, but most certainly as a consequence of the migration processes in the northern and central parts of the country due to the war.^39^

An additional result of interest related to the ITS is that, as seen in previous studies, the longer the observation period following the implementation of the strictest restriction measures, the less noteworthy the reduction in the volume of injuries. Studies conducted at the onset of the pandemic in LMICs tend to be those with the sharpest reductions, regardless of the design.^7 16 18 35^ Confinement measures and compliance with them are not always extensively described in the literature, which makes comparisons difficult. In Mozambique, the measures were very strict during the first phase and lessened thereafter. Not surprisingly, our results suggest that, at their onset, those stringent confinement measures immediately reduced the average weekly number of injuries.

An alternative explanation to the reductions and differences observed between studies pertains to differential “attraction” to the ED of specialized hospitals like the ones included in this study. Referral pathways may have changed due to new priority settings,^16 17 32 35^ in particular during the first restriction period,^47^ or guardians might have been less inclined to take injured children to those large hospitals, for example, due to fear of infection.^7 35^ It must be underlined, however, that the hospitals of the country with the lowest numbers of patients (Quelimane Central Hospital and Nampula Central Hospital) received more injured children in 2020 during the two restrictions periods than compared with the same period the previous year. The numbers were small though compared with the two other hospitals, but higher numbers may be linked to the intensification of war, as the north of the country received many war refugees and orphaned children, and possibly injured ones.

Admissions to the ICU and severe injuries in Mozambique during 2020 increased remarkably despite minor differences between hospitals and restrictions periods. These can be related to the measures implemented during these periods, where people avoid going to hospitals, except for cases that cannot be managed at home or that need specialized (surgery) care, or that put a person’s life in danger. It is also noted that the health system prioritized treatment for COVID-19 and that there were disruptions in healthcare for other diseases or conditions.^47^

### Strengths and limitations

To our knowledge, this is the first study to describe national, multihospital pediatric injury trends during the COVID-19 pandemic in Mozambique. The main strength is that it encompasses the three main regions and involves the four main and largest referral hospitals in the country. Another strength is that data were accessed from a relatively long prepandemic period as well as from the onset of restrictions during 2020, allowing for a robust ITS with weekly data. ITS applied in a quasi-experimental design is an effective and strong way to evaluate the impact of a “crisis” like the COVID-19 pandemic when randomization is not possible.^48^ ITS can detect changes and determine whether those changes are temporary or long-lasting, and can also assess their significance. The method is more sensitive to differences in the effects of the event under study and can be conducted with a small sample size.^44 49^

The first limitation of this study is that there was no national systematic injury surveillance system in place in Mozambique; thus, secondary data in the form of ED records from the participating hospitals were used. These records consist mainly of handwritten records and paper-based patient records stored at the hospital records department, with no backup data. Handwritten materials may entail problems during data compilation, for example, identification of patient numbers or specific injury characteristics. It is also possible that some records were not available at the time of data collection, for example, if data are not stored yet for some reason or if data were lost, which was the case in 2019 for two hospitals: a fire at Maputo Central Hospital in mid-2019 and a flood in March 2019 in Beira Central Hospital. These losses could have led to an underestimation of the number of cases admitted in 2019, and consequently explain the shape of the slope from 2019 to the beginning of 2020.

Another limitation is that the data on hand were based on injury admissions in specialized hospitals and thus may not be representative of what occurred in hospitals at other levels of care in the country, where patients with mild to moderate injuries who did not stay more than 12 hours in the central hospitals may be seen. Indeed, during the COVID-19 pandemic, some children may have been taken for treatment to other healthcare facilities apart from the main central hospitals where the study took place.

The last limitation is that we performed the ITS on data from all hospitals aggregated, whereas two out of the four hospitals, although the smallest ones, had more ED attendances for injury during the restriction periods than during the same period the year before or during the prerestriction period in 2020. Hospital-specific analyses were not feasible due to the small size of data sets of the hospitals.

### Implications

The study provides insight into the changes in the number of pediatric injuries through the first year (2020) of the pandemic in Mozambique. It also shows an association between strictness and the nature of the restrictions and the weekly number of injuries, during which child exposure to most sources of hazards was limited, except in the home environment. These results echo those obtained in both HICs and LMICs and suggest that when restriction measures were most coercive, by and large, in central hospitals, the burden of severe injuries that require specialized care decreased. Whether this reflects a true drop (attributable to reduced exposure) or a hesitancy in the population to present to hospitals or to changed referral patterns between levels of care remains to be determined. Research is needed on whether the hospitals concerned were able to offer the same quality of care as before the pandemic as many resources were reallocated.

Intracountry variations also deserve attention given that the central hospitals in the north and center of the country (Nampula and Quelimane) did not show reductions in the number of injuries. Eventually, this could reflect lower compliance with the restrictions imposed or related to the intensification of the war.

## Conclusion

The restriction measures implemented to control the spread of COVID-19 in Mozambique in April 2020 were associated with an immediate, sharp, and significant reduction in ED attendances for pediatric injuries at the country level, followed by a progressive increase in spite of restrictions. Changes in volume, mechanisms, and severity of pediatric injuries were not only period-specific but also hospital-specific.

## Data Availability

All data relevant to the study are included in the article or uploaded as supplementary information.
